# Effects of Dietary Alteration on the Gut Microbiome and Metabolome of the Rescued Bengal Slow Loris

**DOI:** 10.3389/fmicb.2021.650991

**Published:** 2021-03-24

**Authors:** Qingyong Ni, Chen Zhang, Diyan Li, Huailiang Xu, Yongfang Yao, Mingwang Zhang, Xiaolan Fan, Bo Zeng, Deying Yang, Meng Xie

**Affiliations:** ^1^Farm Animal Genetic Resources Exploration and Innovation Key Laboratory of Sichuan Province, Sichuan Agricultural University, Chengdu, China; ^2^College of Animal Science and Technology, Sichuan Agricultural University, Chengdu, China; ^3^College of Life Science, Sichuan Agricultural University, Yaan, China

**Keywords:** metabolomics, microbiome, short-chain fatty acids, Bengal slow lorises, dietary alteration

## Abstract

Bengal slow lorises (*Nycticebus bengalensis*) are threatened by illegal trade. Subsequently, numerous wild-born individuals are rescued and transferred to rescue centers. Metabonomic analysis of intestinal microbiomes has increasingly played a vital role in evaluating the effects of dietary alteration on the captive status of endangered non-human primates. A synthetic analysis was done to test the differences in gut microbes and fecal metabolites between two dietary groups of Bengal slow lorises across 8 weeks. Dietary interventions led to intra-group convergence and inter-group variation in the composition of intestinal flora, metabolites, and short-chain fatty acids (SCFAs). The control diet, consisting of gums and honey, significantly increased the abundance of some potential probiotics, such as *Bifidobacterium* and *Roseburia*, and the concentration of some anti-disease related metabolites. The decrease in some amino acid metabolites in the original group fed without gums was attributed to poor body condition. Some distinct SCFAs found in the control group indicated the dietary alteration herein was fat-restricted but fiber deficient. Cognizant of this, plant exudates and fiber-enriched food supplies should be considered an optimal approach for dietary improvement of the confiscated and captive Bengal slow lorises.

## Introduction

Metabonomics of intestinal microbiomes is an emerging tool for exploring the effects of the dietary environment on primates for better conservation (Barelli et al., 2015; Stumpf et al., 2016). Many microbial communities in the intestinal tract of primates are crucial for digestion, absorption, metabolism, and immunity (Yatsunenko et al., 2012; Gomez et al., 2016; Visconti et al., 2019). Comparative studies on the composition and function of the gut microbiome are conducive in evaluating the ecology and evolution of non-human primates, including individuals in captivity (Zierer et al., 2018; Ni et al., 2020). Compared to their wild counterparts, the captive primate population may lose some original microorganisms. They are home to a host of the dominant bacteria in the intestines of modern humans and suffer from various health problems (Clayton et al., 2016). The intestinal microorganisms produce numerous metabolites, such as vitamins and short-chain fatty acids (SCFAs), essential for maintaining the dynamic balance of the intestinal microenvironment (Said and Mohammed, 2006; Nicholson et al., 2012; Louis et al., 2014). A high-fat diet can increase the synthesis of arachidonic acid and lipopolysaccharide in feces and a growing pro-inflammatory factor in the plasma (Wan et al., 2019). Some microbial catabolites activate the immune system, enhance intestinal epithelial barrier, stimulate gastrointestinal motility, gastrointestinal hormone secretion, and regulate intestinal microbial compositions (Roager and Licht, 2018). SCFAs are precursors and energy of hosts’ cholesterol, fat, and protein biosynthesis. They play a crucial role in regulating the intestinal microbial activity and host metabolism (Serino, 2019). SCFAs can regulate metabolic sensors to increase oxidative phosphorylation, glycolysis, and fatty acid synthesis, which generate energy and building blocks required for antibody production (Kim et al., 2016).

The slow loris (Nyc*ticebus*, Lorisidae, Primates) is one of the primate taxa most threatened by illegal trade (Ni et al., 2018). It is listed in Appendix I of the Convention on International Trade in Endangered Species of Wild Fauna and Flora (CITES). Numerous animals rescued from the illegal trade are raised in captivity, and some of them are released into the wild (Nekaris and Starr, 2015; Ni et al., 2018). The captive animals experience remarkable changes, especially diet in the living environment. The captive diet is dominated by commercial food, such as fruits and meats (Ni et al., 2020). However, plant secretions such as gums are a staple food of slow lorises in the wild because they are readily available in all seasons (Das et al., 2014; Cabana et al., 2017, 2018). The shift from the wild to the captive diet may be detrimental to the captive animals. For example, Cabana and Nekaris (2015) found that long-term feeding of a diet of “more fruit and fewer plant secretions” led to more oral diseases in captive slow lorises. Incorrect diets may lead to elevated perishability and mortality of the captive individuals and those reintroduced into the wild (Nekaris et al., 2010, 2014).

The conservationists usually recommend the wild dietary supply to diminish the negative effect of captivity. Williams et al. (2015) reported that the slender captive lorises showed less abnormal behavior after adding insects, nectar, and plant secretions to their diet. Metabolomics of the gut microbiome provides an ideal model for understanding the effects of dietary improvement, thereby laying a strong foundation for the successful reintroduction of endangered primate species to the wild (Albenberg and Wu, 2014; Amato et al., 2015; Clayton et al., 2018; Greene et al., 2018; Cabana et al., 2019). This study aimed to explore the characteristics and temporal variation of gut microbiota, fecal metabolites, and SCFAs of captive Bengal Slow Lorises (*Nycticebus bengalensis*) by determining the effects of dietary alterations.

## Methods

### Animal Ethics Statement

Sample collection and experimental protocols were performed in accordance with the Institutional Review Board (IRB13627) and the Institutional Animal Care and Use Committee of the Sichuan Agricultural University, China under permit number DKY-2018302039, as well as Administration for Wild Animal Protection in Yunnan Provinces, China and adhered to the American Society of Primatologists Principles for the Ethical Treatment of Non-Human Primates.

### Study Site and Animals

The study was conducted between June and November 2019 in Dehong Wildlife Rescue Center in Yunnan, China (24.38287° N, 98.45872° E). A total of 42 rescued Bengal slow lorises were separately housed in small iron cages (80^∗^50^∗^60 cm) in the rescue center with the same diet for at least one month before the study. Ten healthy individuals with a similar time budget as study animals were selected after a 2-week pre-observation using a night vision monitoring system (TCNC9401S3E-2MP-I5S and TC-NC9501S3E-2MP-I3S infrared camera, Tiandy Technologies CO., LTD., Tianjin, China). The individuals were assumed to be wild born despite being rescued from various resources because there are no breeding centers of slow lorises worldwide.

### Dietary Supply and Sample Collection

The ten individuals were divided into two groups (*n* = 5). One group, defined as the original group (OG), was fed with the original diet (50 g peeled bananas, 50 g apples, 40 g rice, and 10 g frozen locusts). The others were defined as the control group (CG) which was fed with 19 food types, including fruits (peeled bananas, apples, pears, grapes, dragon fruit, guava, figs, passion fruit), and vegetables (broccoli, lettuce, carrots, cooked pumpkin). They were also fed with animal proteins (live locust, *Zophobas atratus*, *Blaptica dubia*, boiled eggs), soft gums (food grade Arabic gum, wild peach gum), and honey (hundred flowers honey). Their feeding bouts were observed, and their food preference was recorded for 2 weeks. There were four preferred food types in each feeding bout in the control group. The control diet was defined as 50 g peeled bananas, 50 g soft peach gum, 10 g live locusts, and 5 g honey.

Both groups were fed once at 6 pm every day. Their feces were collected at the beginning (0W) and the end of the fourth (4W) and the 8th week (8W) in the 8-week study period. Finally, 30 samples were obtained and categorized into 6 week-groups: OG-0W, OG-4W, OG-8W, CG-0W, CG-4W, and CG-8W. The fecal samples were collected from their enclosures in the morning and maintained in dry ice before storage at −80°C in the laboratory.

### Isolation and DNA Sequencing

The total fecal bacterial DNA was extracted using the PowerSoil DNA Isolation Kit (MO BIO Laboratories, Carlsbad, CA, United States) following the manufacturer’s protocol. The quality and quantity of the DNA were subsequently determined using a NanoDrop spectrophotometer (ND-1000, NanoDrop Technologies, Wilmington, DE, United States). The V3-V4 region of the bacterial 16S-rRNA gene was amplified by polymerase chain reaction (PCR, 98°C for 2 min, followed by 30 cycles of 98°C for 30 s, 50°C for 30 s, 72°C for 60 s, and 72°C for 5 min.) using the primers 338F (5′-ACTCCTACGGGAGGCAGCA-3′) and 806R (5′-GGACTACHVGGGTWTCTAAT-3′).

The V3-V4 region of the archaea 16S-rRNA gene was also amplified by PCR (95°C for 5 min, followed by 35 cycles of 95°C for 30 s, 50°C for 30 s, 72°C for 40 s, and 72°C for 7 min) using the primers Arch349F (5′-GYGCASCAGKCGMGAAW-3′) and Arch806R (5′-GGACTACVSGGGTATCTAAT-3′). The PCR products were mixed with an equal volume of 2 × loading buffer and electrophoresed in a 1.8% agarose gel for detection. Samples with a band at approximately 450 bp were mixed in equidensity ratios followed by purification using a GeneJET Gel Extraction Kit (Thermo Fisher Scientific, Waltham, MA, United States). Sequencing libraries were validated using an Agilent 2100 Bioanalyzer (Agilent Technologies, Palo Alto, CA, United States) and quantified with a Qubit 2.0 Fluorometer (Thermo Fisher). Finally, paired-end sequencing was conducted on an Illumina HiSeq 2500 platform (Illumina, Inc., San Diego, CA, United States) by the Biomarker Bioinformatics Technology Co., Ltd. (Beijing, China).

### Metabolomic Analysis of the Fecal Samples

The frozen samples were thawed on ice. A 50 mg aliquot of each sample was dispensed into Eppendorf tubes, and 500 μL of precooled glacial methanol (containing 1 μg/mL of 2-chlorophenyl alanine as the internal standard) added, followed by vortexing for 1 min to mix well. The mixture was centrifuged at 12,000 rpm for 10 min at 4°C, and the supernatant was used for liquid chromatography-tandem mass spectrometry (LC-MS/MS) analysis.

LC-MS/MS analysis was conducted using an ultra-high-performance liquid chromatography - local track quality (UHPLC-LTQ) Orbitrap at a column temperature of 40°C. The equipment was equipped with a 100 mm × 2.1 mm, 1.8 μm Acquity UPLC HSS T3 C18 (Waters, Milford, MA, United States). The mobile phase contained: ultrapure water (0.04% acetic acid), acetonitrile (0.04% acetic acid); elution gradient: 0 min water/acetonitrile (95:5 V/V), 11.0 min 10:95 V/V, 12.0 min 10:90 V/V, 14.0 min 95:5 V/V; flow rate 0.4 mL/min; and injection volume 2 μL. The primary MS conditions were electrospray ionization, temperature 500°Cmass spectrum voltage 5,500 V (positive), −4,500 V (negative), ion source gas I 55 psi, gas II 60 psi, gas curtain gas (CUR 25 psi), and collision-induced ionization on High. In the triple quadrupole (Qtrap), each ion pair was scanned based on the optimized de-clustering potential S and collision energy.

### Measurement of SCFAs

Twenty milligrams of the fecal sample were mixed with 1 mL of phosphoric acid solution (0.5% v/v), homogenized, and subjected to ultrasound for 5 min in an ice bath. The suspension (0.02 mL) was then collected in an Eppendorf tube and mixed with 1 mL of methyl tert-butyl ether (MTBE) solution containing 0.3 mg/L 2-methyl valeric acid as the internal standard. The mixture was then vortexed for 3 min, ultrasonicated for 5 min in an ice bath, and then centrifuged at 12,000 rpm for 10 min. The supernatants (0.5 mL) were subsequently collected in a glass autosampler vial and mixed with an equal volume of MTBE solution containing 0.3 mg/L of 2-methyl valeric acid as the internal standard.

Gas chromatography-tandem mass spectrometry (GC-MS/MS) was performed on a DB-FFAP capillary column (30 m × 0.25 mm × 0.25 μm) with high purity helium at a flow rate of 1.2 mL/min. The front injection temperature was 200°C. The GC-MS/MS analysis was 95°C hold on for 1min, raised to 100°C at a rate of 25°C/min, raised to 130°C at a rate of 17°C/min, hold on for 0.4 min, raised to 200°C at a rate of 25°C/min, hold on for 0.5 min, after running for 3 min. The transfer line and ion source temperature were maintained at 230°C. The detector operated in a multiple reaction monitoring scan mode with an ionization voltage of 70 eV.

### Statistical Analysis

The raw fastq data were demultiplexed based on their barcodes, and the paired-end reads for all the samples run through Trimmomatic (V. 0.35)^1^ to remove the low-quality base pairs. FLASH (V. 1.2.7)^2^ was used to join the paired reads and sequences over a 50-bp sliding window. Sequences with an average base quality score lower than 20 were removed, while sequences with overlapping lengths (>10 bp) were retained. The demultiplexed reads were clustered with a 97% similarity cutoff using UPARSE (V. 10.0)^3^ to generate operational taxonomic units (OTUs). Taxonomic classification of the representative OTUs sequences was done using the Silva 132 database^4^. The microbial diversities were analyzed using the free online Biomarker Cloud Platform^5^. The alpha diversity indices Shannon, Simpson, and Chao 1 were calculated using mothur (V. 1.30)^6^ and then plotted using R (V. 3.2.3)^7^. Alpha diversity differences between groups were analyzed using the t-test by IBM SPSS Statistics (V. 23.0)^8^. The Bray-Curtis distance matrices were calculated using mothur and visualized by principal coordinate analysis (PCoA). The analysis of similarities (ANOSIM) was used to determine the differences in the gut microbiota abundance between the two groups. Linear discriminant analysis (LDA) effect size (LEfSe) was used to identify the differential biomarkers between the groups; an LDA cutoff score larger than 4.0 was statistically significant.

The widely discriminant metabonomics analysis was carried out using Analyst (V. 1.6.3)^9^ to process the MS data. The metabolites were compared with those in the existing self-built database to identify them (Du et al., 2020) based on the Q1 and Q3 information of the metabolites. Q1 was the mass charge ratio of the parent ion and Q3 the mass charge ratio of the fragment ion. The chromatographic peaks were integrated and corrected using the Multi Quanta software (V. 3.02, See Text Footnote 9). The principal component analysis was carried out to determine the degree of inter-and intra-group variations. The metabolites of different varieties or tissues were preliminarily screened from the variable importance in projection (VIP) based on the orthogonal partial least squares’ discriminant analysis (OPLS-DA) model and ANOSIM. The metabolites with the highest VIP values are considered to be the most discriminative and typically, VIP ≥ 1 are significant. The differential metabolites were further screened by univariate analysis of the *P*-value and multiple differential values (Fold Change, FC).

Short-Chain Fatty Acidss quantification was done by measuring the peak areas for acetic acid, propionic acid, butyric acid, isobutyric acid, valeric acid, and isovaleric acid relative to 2-methyl valeric acid. The FC values of SCFAs was calculated, and the t-test was used to calculate the *P*-value. The Spearman rank correlation coefficient was used to analyze the correlation between the abundance of the gut microbiome (family) and metabolites. Distinct metabolites with a coefficient greater than 0.7 and a *P*-value less than or equal to 0.05 were selected and plotted. A sub-network map was used to show the correlation between the distinct microorganisms and metabolites at the family level.

## Results

### Gut Microbiota and Archaea

**FIGURE 1:**
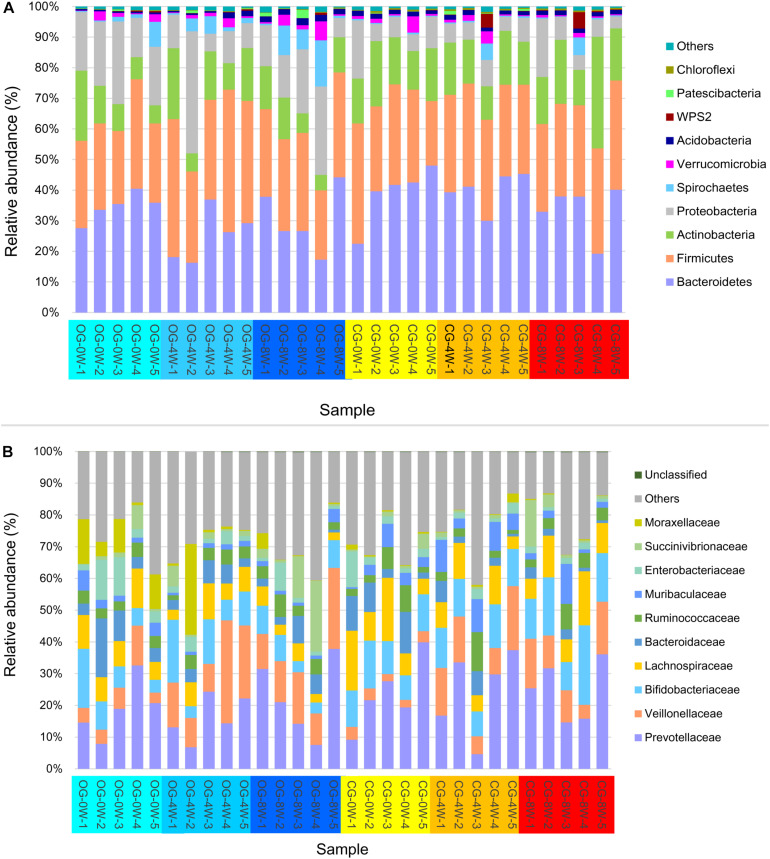
Relative abundance of intestinal bacterial taxa in different week-groups on the phylum **(A)** and family level **(B)**.

A total of 1,993,828 valid sequences corresponding to 66,461 ± SD 1778 (ranging between 63,195 and 70,420) sequences for each Bengal slow loris were obtained from the 30 fecal samples. Bacteroidetes (33.83% ± SD 9.03%), Firmicutes (31.73% ± SD 5.72%), Actinobacteria (14.51% ± SD 6.49%), and Proteobacteria (12.62% ± SD 8.69%) were the most abundant phyla in the gut microbiome of both groups throughout the study period (Figure 1A). The predominant bacterial families isolated from the fecal samples at the initial week were Prevotellaceae (21.25% ± SD 9.45%), Lachnospiraceae (10.39% ± SD 4.97%), and Bifidobacteriaceae (10.02% ± SD 4.20%). However, the relative abundance of Veillonellaceae significantly increased in the 8th week in the original (15.12% ± SD 6.28%) and control groups (11.40% ± SD 4.91%) (Figure 1B).

**FIGURE 2:**
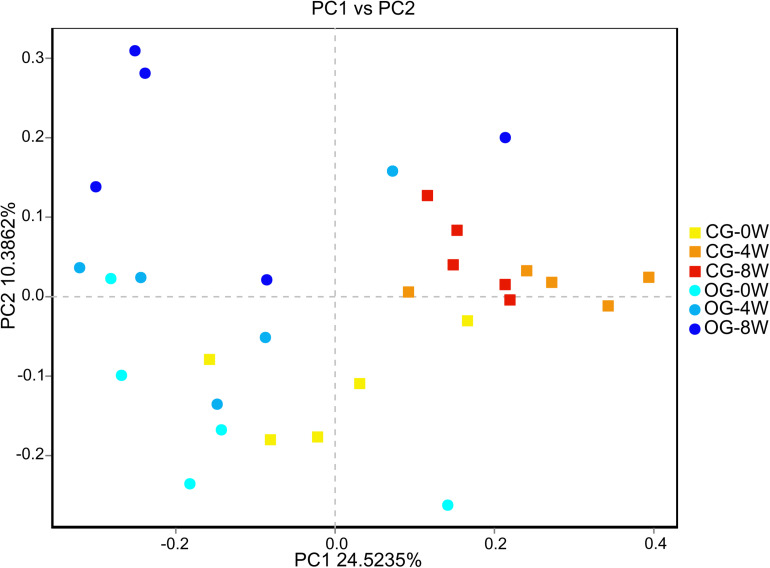
PCoA-analysis based on Bray-curtis dissimilarities for the bacterial microbiota in different week-groups of Bengal slow lorises.

**FIGURE 3:**
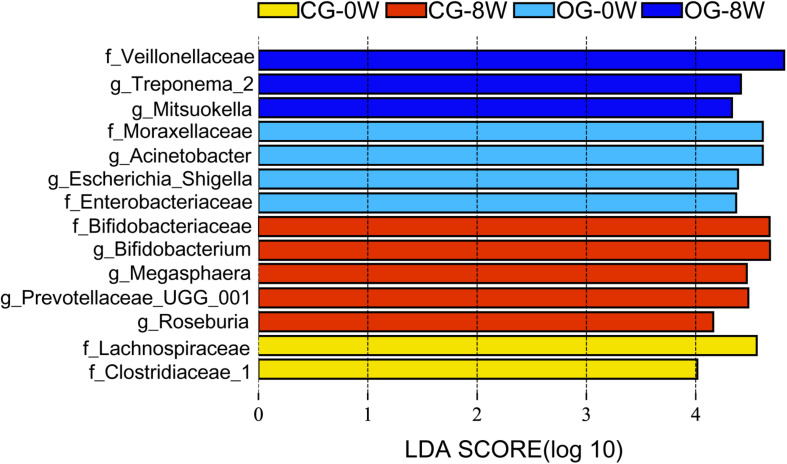
Differences in intestinal bacterial taxa among week-groups determined by LEfSe analysis. The highlighted taxa were significantly enriched in the group that corresponded to each color. LDA scores can be interpreted as the degree of difference in relative abundance. Family and genus are indicated by *f* and *g* respectively.

There were significant differences in ace and Chao 1 indexes between OG-0W and CG-0W (ace: *t* = −4.878, *df* = 7, *P* = 0.002; Chao 1: *t* = −2.672, *df* = 7, *P* = 0.032). In addition, there were significant differences between OG-0W and OG-8W (ace: *t* = −3.858, *df* = 8, *P* = 0.005; Chao 1: *t* = −2.431, *df* = 7, *P* = 0.047) (Supplementary Figures 1A,B). However, there were no significant differences in Shannon and Simpson indexes among the different week-groups (Supplementary Figures 1C,D). Based on the PCoA Bray-Curtis dissimilarity analysis, the OTU level’s microbial composition appeared to be similar between the two groups in the initial week but gradually differed in the fourth and 8th week (*r* = 0.402, *P* = 0.001; Figure 2). LEfSe analysis revealed eight genera and six families were distinct in 4 week-groups (Figure 3 and Supplementary Figure 2). CG-8W had a relative abundance of genera *Bifidobacterium*, *Megasphaera*, *Prevotellaceae_UCG_001*, and *Roseburia* (Supplementary Figures 3A–D). On the other hand, OG-8W had a higher abundance of genera *Mitsuokella*, *Treponema*, and family Veillonellaceae (Supplementary Figures 3E,F).

**FIGURE 4:**
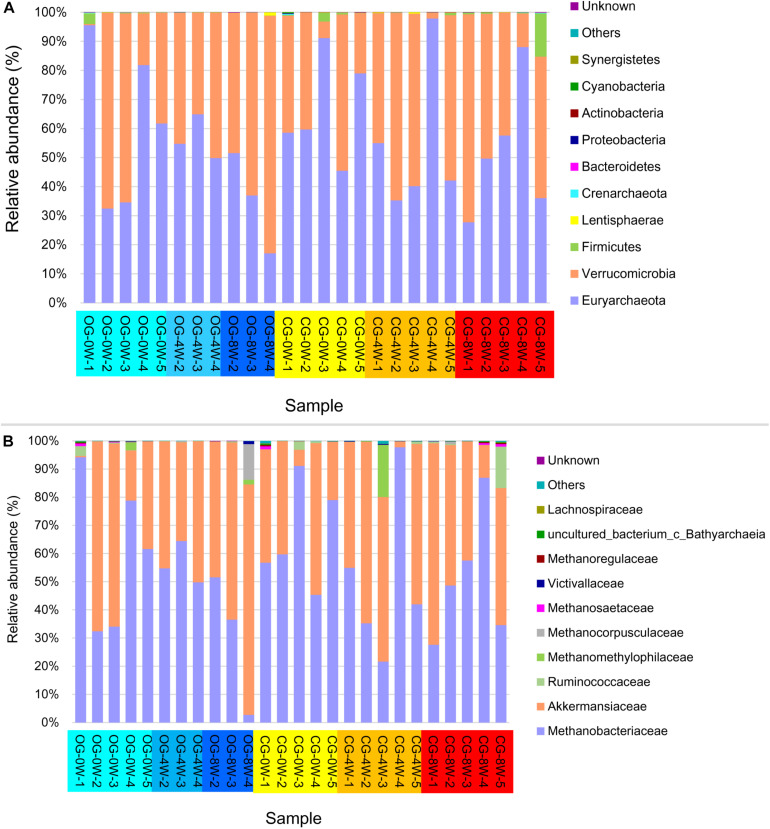
Relative abundance of intestinal archaea taxa in different week-groups on the phylum **(A)** and family level **(B)**.

The archaea showed a similar composition pattern between the different groups (Figure 4). Euryarchaeota (55.55% ± SD 21.97%) and Verrucomicrobia (43.23% ± SD 22.01%) were the most dominant at the phyla level archaea in all the individual samples. Methanobacteriaceae (53.82% ± SD 23.66%) and Akkermansiaceae (43.20% ± SD 21.10%) dominated the family level. The abundance of methanogens (Methanobacteriales, Methanomicrobiales, and Methanosarcinales) at 0W and 8W did not vary significantly in the control group (*t* = 1.613, *df* = 6, *P* = 0.160). Besides, they did not vary significantly at 0 W and 8 W in the original group (*t* = 1.133, *df* = 8, *P* = 0.292). However, the abundance of methanogens in the control group was higher than that in the original group at 8W though the difference was not significant (*t* = −1.613, *df* = 5, *P* = 0.294).

### Fecal Metabolomics Profiles

**FIGURE 5:**
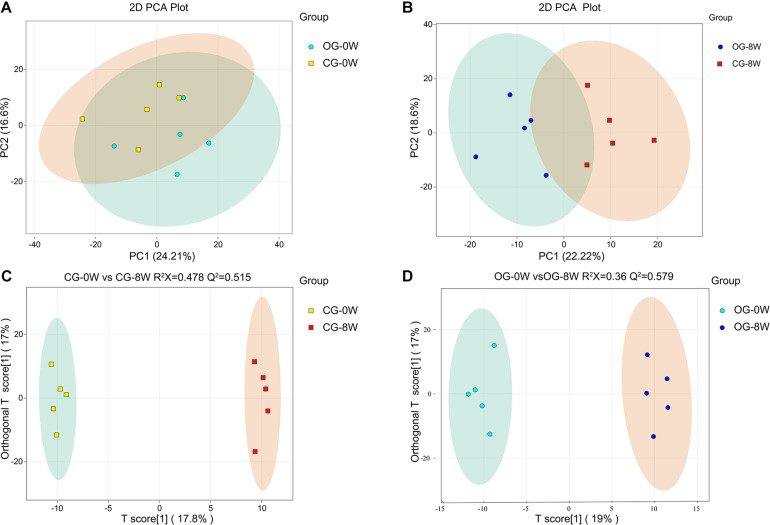
PCA-analysis on total fecal metabolites at the beginning (0W) **(A)** and the 8th week (8W) **(B)** between the two dietary groups. The OPLS-DA score plot of CG-0W vs. CG-8W **(C)** and OG-0W vs. OG-8W **(D)**.

There were 621 compounds positively identified through LC-MS/MS analysis. Organic acids and their derivatives (20.77% of total identified compounds) were the most dominant, followed by amino acids (15.46%), nucleotides (9.33%), benzene and substituted derivatives (8.37%), and carbohydrates (6.76%). No significant differences were found in the composition of fecal metabolites between the groups at 0W (OG-0W vs. CG-0W: *P* = 0.638). However, there were significant shifts after diet intervention (OG-8W vs. CG-8W, *P* = 0.011) (Figures 5A,B). The OPLS-DA model revealed that the fecal metabolites of the two groups could be significantly distinguished at different periods. R^2^X and Q^2^ values indicated that both models were of high quality (Figures 5C,D). There were 125 distinct metabolites at 8W of the original group compared to 0W (Supplementary Table 1), mainly grouped into organic acid and their derivatives (24.80%), amino acids (23.20%), and benzene and substituted derivatives (8.80%).

**FIGURE 6:**
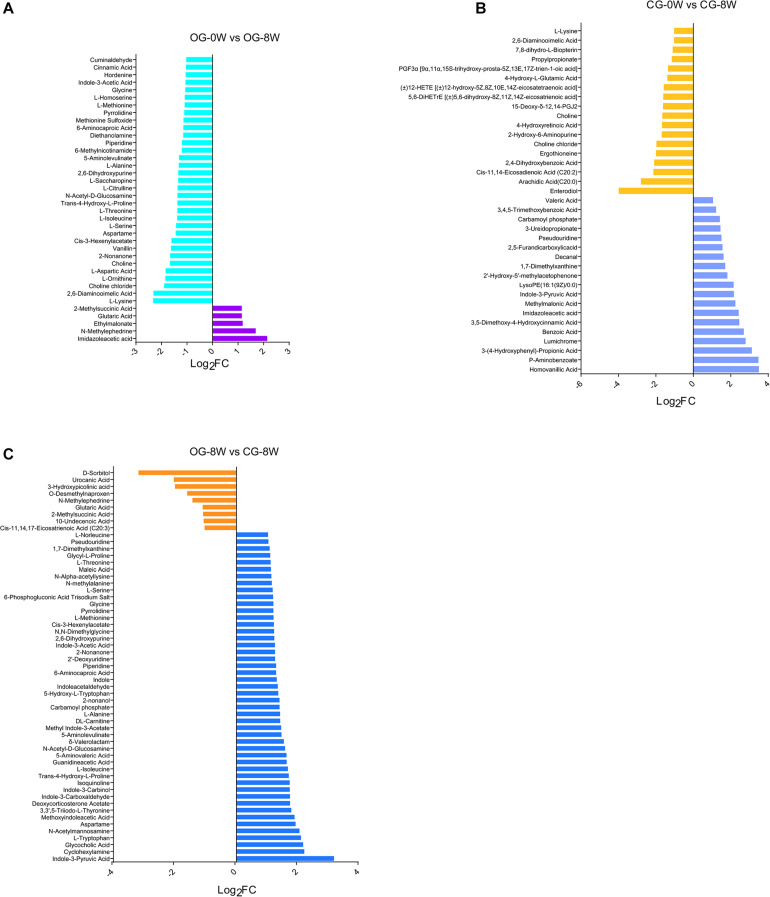
Bar-chart of the distinct metabolites between week-groups based on Log_2_FC value: **(A)** OG-0W and OG-8W; **(B)** CG-0W and CG-8W; **(C)** OG-8W and CG-8W.

There were 38 fecal metabolites significantly changed in the original group after 8 weeks. Among them, 13 amino acids, including 1-lysine, l-threonine, l-ornithine, l-aspartic acid, and l-serine, were decreased (Figure 6A and Supplementary Table 1). Individual metabolite analysis of the control group indicated that the contents of 18 metabolites had decreased. The metabolites included the enterodiol, 12-HETE (± 12-hydroxy-5Z, 8Z, 10E, 14Z-eicosatetraenoic acid), and two long-chain fatty acids: arachidic acid (C20:0) and Cis-11, 14-Eicosadienoic acid (C20:2). However, there were 19 metabolites whose relative abundance had increased. They included the homovanillic and p-aminobenzoic acid and 3,5-dimethoxy-4-hydroxycinnamic acid (sinapic acid) (Figure 6B and Supplementary Table 2). Besides, there were 61 distinct fecal metabolites between OG-8 and CG-8, including amino acids and organic acid indole and their derivatives (Figure 6C and Supplementary Table 3).

### SCFA Profiles

Acetic acid (3.00 ± SD 1.44 ug/mg) was the most dominant SCFAs in all the individual samples at 0 W. It was followed by propionic acid (0.77 ± SD 0.29 ug/mg), butyric acid (0.56 ± SD 0.48 ug/mg) and valeric acid (0.12 ± SD 0.11 ug/mg) (Supplementary Table 4). However, there was a significant increase in isovaleric acid at 8 W compared to 0 W in the control group (*t* = −2.532, *df* = 5, *P* = 0.049). A significant increase in propionic acid was observed in the original group (*t* = −2.857, *df* = 6, *P* = 0.031) (Table 1). Based on the FC value, the abundance of seven SCFAs in CG-8W was lower than that in OG-8W though the differences were insignificant (Table 1).

**TABLE 1:** Analysis in the concentration of SCFAs between week-groups based on *T*-test and Fold Changes (FC) value.

Compounds	OG-0W vs OG-8W		CG-0W vs CG-8W		OG-8W vs CG-8W	
			
	*P*	FC	*P*	FC	*P*	FC
Acetic acid	0.159	1.538	0.236	0.624	0.170	0.602
Propionic acid	0.049*	2.039	0.814	1.080	0.146	0.610
Isobutyric acid	0.293	1.533	0.112	1.664	0.468	0.791
Butyric acid	0.214	2.326	0.942	1.054	0.334	0.514
Isovaleric acid	0.301	1.573	0.031*	2.259	0.950	0.981
Valeric acid	0.054	3.630	0.296	1.827	0.110	0.424
Caproic acid	0.172	4.500	0.796	0.860	0.427	0.569

*The P values less than 0.05 are in bold.

### Microbiome-Metabolome Associations

**FIGURE 7:**
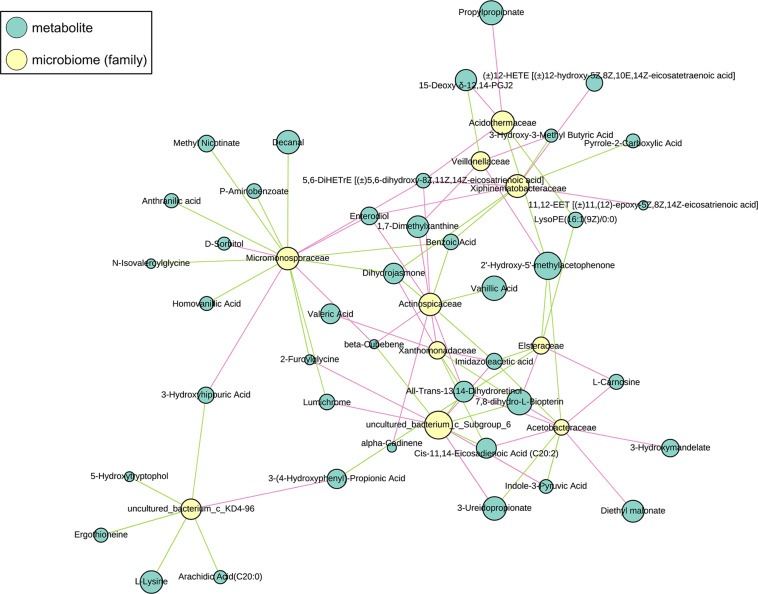
Sub-network view of relationships between intestinal metabolites and gut microbiome composition in the control group of captive Bengal slow lorises. Yellow and blue nodes represent bacteria and metabolites respectively. Node size represents the number of connections of a given taxa or metabolite within the network. The correlation between them is represented by line segments. The red and green segments represent positive and negative correlation respectively.

The sub-network map between CG-0W and CG-8W was categorized into three branches (Figure 7). The first branch showed the numerous correlations between Acetobacteraceae, Acidothermaceae, Asteraceae, Veillonellaceae, Xanthomonadaceae, Xiphinematobacteraceae, uncultured_bacterium_c_Subgroup_6, and amino acids. It also showed numerous correlations between benzene and its substituted derivatives, coenzymes and vitamins, fatty acyls, lipids, fatty acids, organic acid and their derivatives, oxidized lipid, and pteridines and their derivatives. The second branch showed the relationship between Micromonosporaceae and some metabolites (mainly benzene and its substituted derivatives, organic acid and their derivatives, amino acid). The remaining branch showed the correlation between uncultured_bacterium_c_KD4-96 and arachidic acid, l-lysine, ergothioneine, 5-hydroxytryptophol, 3-hydroxyhippuric acid, and 3-(4-hydroxyphenyl)-propionic acid.

## Discussion

Herein, all individuals were assumed to be wild-born because of the lack of Bengal slow lorises breeding centers in China and the surrounding nations. The individuals were rescued from different sites and housed in captivity for varied periods. These variations were postulated to explain the significant differences in intestinal microbiota diversity among the individuals initially. Gut microbiota can quickly and appropriately change their functions to cope with diet changes, thereby improving the utilization of calories and nutrients provided by readily available plant foods (David et al., 2014). The diversity of intestinal microorganisms were almost similar within the original or control group during the 8-week dietary intervention because of the constant food supply. However, the microbial relative abundances between the two groups were different. Faith et al. (2011) revealed that shifting dietary macronutrients of inbred mice broadly and consistently altered the gut microbiome within a single day. Different diets caused by varying geographical locations or seasons can lead to significant differences in gut microbiota among populations of non-human primates and convergence of differences among populations because of food restrictions (Gomez et al., 2015, 2016; Greene et al., 2018).

Cabana et al. (2019) reported that *Actinobacteria* was the most represented phylum in wild Java slow loris (*N. javanicus*) in the captive group. Another study suggested that *Bacteroidetes*, *Firmicutes*, and *Proteobacteri* dominated the gut microbiota of captive Bengal Slow Lorises (Ni et al., 2020). Herein, there was a higher relative abundance of *Bacteroidetes* and fewer *Actinobacteria*, consistent with their captive status, particularly the diet dominated by commercial food. Besides, there were significant differences in intestinal microbial composition between the two groups because of the dietary alteration. Notably, *Bifidobacterium* and *Roseburia*, which are essential potential probiotics, were more abundant in the individuals fed with the control diet. *Bifidobacterium* plays a role in improving the epithelial barrier function, secretion of inhibitory substances such as bacteriocins and hydrogen peroxide, immunomodulation, inhibition of expression of virulence factors, and competitive exclusion-possibly through colonization resistance (Rea et al., 2013). The genus *Roseburia* are Gram-positive bacteria that produce butyrate during fiber fermentation, increasing energy uptake and utilization by the host epithelium and modulating local inflammation and cell repair through apoptosis (Canani et al., 2011). However, butyrate production may be restricted due to the scarcity of dietary fiber in the diet of captive individuals (Liu et al., 2018). The abundance of methanogenic archaea was also higher in the control group compared to the original group. Cabana et al. (2019) suggested that the increasing methanogenic archaea in wild slow lorises were attributed to the improved diet dominated by plant exudates, fibrous plants, and insects.

Plant exudates such as tree gums comprise more than 80% of the wild Bengal slow lorises diet (Cabana and Nekaris, 2015). However, the rescue centers did not provide any plant exudates for the captive individuals across China, leading to a potential differentiation in the intestinal microorganisms between wild and captive populations (Ni et al., 2020). Peach gum is a natural polysaccharide gum with poor water-solubility. It promotes the growth of intestinal potential probiotics such as *Bifidobacterium* and *Lactobacillus* (Gu et al., 2013), thereby increasing probiotic abundance in the gut microbiota of the slow lorises. The lack of gums is associated with the decrease of *Prevotella* abundance in the captive slow lorises (Cabana et al., 2019; Ni et al., 2020). Herein, the abundance of *Prevotella* did not vary significantly among groups. However, the abundance of *Prevotellaceae_UCG_001* increased significantly in the Bengal slow lorises fed with peach gum. *Prevotellaceae_UCG_001* is an indicator of dietary alteration (Zhang et al., 2018). High-fat diets reduce the relative abundance of *Prevotellaceae_UCG-001* in the cecum of mice. However, polysaccharide intervention can significantly repair this difference (Huang, 2019).

Significant variations also occurred in the fecal metabolite composition of captive Bengal slow lorises after dietary intervention. Some of the metabolites play critical roles in predicting individual health status. For example, the unconjugated homovanillic acid, which was the most increased metabolite in the control group, is the final product of dopamine metabolism (Bacopoulos et al., 1978). Wang et al. (2020) reported that children with autism spectrum disorders had decreased homovanillic acid levels, which increased after treatment. Another distinct metabolite was sinapic acid, which is associated with the nutritional and biological functions of honey (Zhao et al., 2017). The relatively high abundance of sinapic acid in the control group metabolites resulted from the honey supply in the diet. Nonetheless, it is widely found in plants, spices, fruits, vegetables, grains, and oil crops (Thiel et al., 2015). It is active in scavenging free radicals, thus inhibiting lipid peroxidation, anti-cancer, inflammation, and anxiety (Ma et al., 2016). It also has antibacterial activity against harmful bacteria (Wang and Ellis, 1998). The decrease of the metabolite Ergothioneine in the control group was attributed to the lack of rice. It is a natural and rare amino acid that the animal body cannot synthesize but can only be absorbed from food (Li and Zhou, 2010).

Moreover, some of the distinct metabolites after dietary alterations may be predictors of disease or ailment. For example, 12-HETE plays an essential role in the development of many diseases, such as cancer, diabetes, and hypertension. It is involved in the occurrence and development of pathological processes such as inflammation and oxidative stress (Cheng et al., 2019). It is also a metabolite of arachidonic acid associated with the high-fat diet (Gross et al., 2005; Wan et al., 2019). The occasional supply of meat or poultry in the husbandry centers may cause a higher relative abundance of 12-HETE for the captive Bengal slow loris. The captive animals could hardly move in the small cages, restricting their fat metabolism (Cheng et al., 2019). L-lysine is an essential amino acid for human beings and animals. It is widely used as a commercial food additive (Jakobsen et al., 2009; Zhang, 2012). Lu et al. (2011) reported that the total amount of lysine and other amino acids were higher in rhesus monkeys with normal hair than depilated individuals. The captive animals may suffer from depilation because of the long-term commercial diet. A food supply containing honey and peach gum is, therefore, an optimal approach for dietary improvement.

SCFAs are mainly produced through the fermentation of indigestible carbohydrates (e.g., cellulose) by anaerobic microorganisms. Therefore, these microorganisms are the medium between gut microbiota and host metabolism and are indicators of the probiotic effects (Serino, 2019). The captive individuals fed with high-fiber food produce more SCFAs to cope with energy demand than the wild free-eating primates (Amato et al., 2015; Greene et al., 2018). In contrast, the lower fecal concentration of SCFAs in the control group of Bengal slow lorises may result from the lack of apples in their diet. Apple pomace significantly contributes to the fiber content for captive animals (Sudha et al., 2007). Nonetheless, it is highly recommended to find an alternative fiber-enriched food resource for the captive slow lorises, such as plant leaves (Das et al., 2014). Long-term feeding of large amounts of fruit may lead to oral diseases (Cabana and Nekaris, 2015).

## Conclusion

Dietary interventions may lead to intra-group convergence and inter-group variations in intestinal flora composition, metabolites, and SCFAs in the captive Bengal slow lorises. Diet alterations characterized by the supply of gums and honey increased the relative abundance of some potential probiotics and anti-disease related metabolites, which had positive effects on the health of the captive individuals. However, some distinct SCFAs identified in the control group indicated that the dietary alterations tended to be fat-restricted but short of fiber. Given the growing number of individuals rescued from illegal trade, more optimal diet designs for the rescued Bengal slow lorises should be developed. As the most essential food source of wild slow lorises, particularly, gum-based diet supply is critical for the husbandry management of this endangered primate taxa.

## Data Availability Statement

The data presented in the study are deposited in the National Genomics Data Center (NGDC), accession numbers CRA003986 and CRA003988.

## Ethics Statement

The animal study was reviewed and approved by the Institutional Animal Care and Use Committee of the Sichuan Agricultural University.

## Author Contributions

QN conceived and designed the experiments, performed the experiments, analyzed the data, prepared figures and/or tables, and authored, or reviewed drafts of the manuscript. CZ performed the experiments, analyzed the data, contributed reagents, materials, and analysis tools, and prepared figures and/or tables. MX performed the experiments and analyzed the data. DL, HX, YY, MZ, XF, BZ, and DY analyzed the data, contributed reagents, materials, and analysis tools. All authors contributed to the article and approved the submitted version.

## Conflict of Interest

The authors declare that the research was conducted in the absence of any commercial or financial relationships that could be construed as a potential conflict of interest.

## Footnotes

1 http://www.usadellab.org/cms/?page=trimmomatic

2 http://ccb.jhu.edu/software/FLASH/

3 https://drive5.com/uparse/

4 https://www.arb-silva.de

5 www.biocloud.net

6 https://www.mothur.org/

7 https://www.r-project.org

8 https://www.ibm.com/cn-zh/analytics/spss-statistics-software

9 https://sciex.com/content/SCIEX/na/us/en.html
