# Supplementary material for: Effects of Dietary Alteration on the Gut Microbiome and Metabolome of the Rescued Bengal Slow Loris
Source: Front Microbiol. 2021 Mar 24;12:650991. doi: 10.3389/fmicb.2021.650991 (PMC8024692; doi:10.3389/fmicb.2021.650991)
Supplement: Supplementary file 1 [file Data_Sheet_1.docx]

**Table S1.** Summary of the distinct metabolites between OG-0W and OG-8W

| Compounds | VIP | FC | Log_2_FC | p | Type |
| --- | --- | --- | --- | --- | --- |
| L-Methionine | 2.076330191 | 0.469684846 | -1.090235048 | **0.000** | down |
| L-Serine | 2.017386321 | 0.371771922 | -1.42751028 | **0.001** | down |
| L-Homoserine | 1.91549211 | 0.470871862 | -1.086593581 | **0.002** | down |
| L-Alanine | 1.959483668 | 0.400153659 | -1.321373994 | **0.003** | down |
| L-Threonine | 1.955969409 | 0.384257942 | -1.379853017 | **0.003** | down |
| L-Saccharopine | 2.066662302 | 0.393544682 | -1.34540065 | **0.003** | down |
| Pyrrolidine | 1.851107976 | 0.464745576 | -1.105486964 | **0.003** | down |
| 6-Aminocaproic Acid | 1.862945075 | 0.453152497 | -1.14193146 | **0.007** | down |
| 2,6-Diaminooimelic Acid | 1.85275693 | 0.201178442 | -2.313452375 | **0.007** | down |
| 5-Aminolevulinate | 1.908900388 | 0.403001756 | -1.311141969 | **0.008** | down |
| 2,6-Dihydroxypurine | 1.774679321 | 0.394794569 | -1.340825953 | **0.008** | down |
| Methionine Sulfoxide | 1.740634537 | 0.455598296 | -1.134165743 | **0.009** | down |
| L-Aspartic Acid | 1.795565903 | 0.279898536 | -1.837024153 | **0.009** | down |
| L-Isoleucine | 1.886494399 | 0.383324281 | -1.383362707 | **0.010** | down |
| Piperidine | 1.836507056 | 0.437185502 | -1.193682536 | **0.010** | down |
| L-Citrulline | 1.690970772 | 0.387404137 | -1.368088733 | **0.010** | down |
| 2-Nonanone | 1.824573089 | 0.316100465 | -1.661544938 | **0.013** | down |
| Trans-4-Hydroxy-L-Proline | 1.835335143 | 0.385214828 | -1.376264856 | **0.014** | down |
| Vanillin | 1.88201883 | 0.323477106 | -1.628264484 | **0.014** | down |
| Cis-3-Hexenylacetate | 1.787426433 | 0.32837673 | -1.606576197 | **0.015** | down |
| 2-Methylsuccinic Acid | 1.836838941 | 2.214293765 | 1.146846634 | **0.016** | up |
| Glutaric Acid | 1.828717907 | 2.219335758 | 1.150127946 | **0.016** | up |
| Ethylmalonate | 1.842419907 | 2.270865888 | 1.183242507 | **0.017** | up |
| Imidazoleacetic acid | 2.006533716 | 4.418211455 | 2.143462468 | **0.017** | up |
| 6-Methylnicotinamide | 1.638278118 | 0.435256733 | -1.200061481 | **0.019** | down |
| Cuminaldehyde | 1.551541125 | 0.489357276 | -1.031039945 | **0.024** | down |
| Aspartame | 1.531216983 | 0.367611105 | -1.443747748 | **0.024** | down |
| Diethanolamine | 1.72286117 | 0.452143487 | -1.145147413 | **0.025** | down |
| Choline chloride | 1.715445794 | 0.268975261 | -1.894454608 | **0.025** | down |
| Cinnamic Acid | 1.561671169 | 0.486432866 | -1.039687386 | **0.025** | down |
| Glycine | 1.840524616 | 0.477761933 | -1.065636186 | **0.026** | down |
| Hordenine | 1.498544763 | 0.482889195 | -1.050235912 | **0.032** | down |
| L-Lysine | 1.748915319 | 0.198503508 | -2.332763589 | **0.037** | down |
| N-Acetyl-D-Glucosamine | 1.631732437 | 0.385798232 | -1.374081565 | **0.038** | down |
| Indole-3-Acetic Acid | 1.433156363 | 0.479913689 | -1.05915313 | **0.042** | down |
| Choline | 1.635189655 | 0.314730377 | -1.667811662 | **0.044** | down |
| N-Methylephedrine | 1.100337277 | 3.232780051 | 1.692775354 | **0.047** | up |
| L-Ornithine | 1.447196068 | 0.278133202 | -1.846152117 | **0.048** | down |
| 3,4,5-Trimethoxycinnamic Acid | 1.253584609 | 3.93181798 | 1.975196535 | 0.050 | up |
| N-Alpha-acetyllysine | 1.544352301 | 0.403138871 | -1.3106512 | 0.052 | down |
| Cis-11,14-Eicosadienoic Acid (C20:2) | 1.328092521 | 2.644208832 | 1.402836121 | 0.052 | up |
| 5-Hydroxyhexanoic Acid | 1.079758057 | 2.365236584 | 1.241984497 | 0.053 | up |
| Compounds | VIP | FC | Log_2_FC | p | Type |
| Orotic Acid | 1.792593751 | 0.141215277 | -2.824031923 | 0.055 | down |
| O-Desmethylnaproxen | 1.670982759 | 2.257701577 | 1.174854803 | 0.059 | up |
| L-Histidine | 1.702764104 | 0.264193349 | -1.920333945 | 0.059 | down |
| N-Acetylmannosamine | 1.467965174 | 0.345821346 | -1.53190117 | 0.061 | down |
| Indoleacetaldehyde | 1.314928899 | 0.480586275 | -1.057132648 | 0.061 | down |
| D-Glucono-1,5-Lactone | 1.548499387 | 0.458176155 | -1.126025716 | 0.061 | down |
| Dodecanedioic Aicd | 1.599042369 | 3.264834085 | 1.707009677 | 0.061 | up |
| N-methylalanine | 1.418228979 | 0.475385836 | -1.072829175 | 0.061 | down |
| Indole | 1.35659229 | 0.497635589 | -1.006838431 | 0.061 | down |
| Dethiobiotin | 1.504546816 | 4.890186059 | 2.289889357 | 0.067 | up |
| L-Tryptophan | 1.301962536 | 0.244859868 | -2.029971757 | 0.068 | down |
| Marmesin | 1.344060055 | 2.079336756 | 1.056123427 | 0.069 | up |
| 5-Hydroxy-L-Tryptophan | 1.326916959 | 0.477528793 | -1.066340369 | 0.072 | down |
| Maltotriose | 1.538589447 | 0.321728206 | -1.636085673 | 0.075 | down |
| 5-Aminovaleric Acid | 1.089459597 | 0.48629635 | -1.040092333 | 0.076 | down |
| Biotin | 1.348203976 | 2.202414804 | 1.139086212 | 0.076 | up |
| Guanidineacetic Acid | 1.085030778 | 0.485095364 | -1.043659704 | 0.076 | down |
| 4-Acetamidobutyric Acid | 1.080854534 | 2.225581114 | 1.154182082 | 0.080 | up |
| L-Arabitol | 1.691729934 | 0.215748716 | -2.21257612 | 0.084 | down |
| (±)5-HETE [(±)5-hydroxy-6E,8Z,11Z,14Z-eicosatetraenoic acid] | 1.587576891 | 0.260577098 | -1.940217801 | 0.086 | down |
| 1-Naphthylacetic Acid | 1.458182168 | 0.40272741 | -1.312124427 | 0.089 | down |
| N6-Succinyl Adenosine | 1.25345299 | 0.181591771 | -2.461229264 | 0.089 | down |
| 4-(Aminomethyl)-5-(Hydroxymethyl)-2-Methylpyridin-3-Ol | 1.303618245 | 0.379906004 | -1.396285582 | 0.090 | down |
| Nicotinamide | 1.051147136 | 6.702681628 | 2.744738409 | 0.090 | up |
| Lysopc 20:1 | 1.451900774 | 0.364568072 | -1.455739876 | 0.091 | down |
| DHA [4Z,7Z,10Z,13Z,16Z,19Z-docosahexaenoic acid] | 1.404144673 | 0.205672402 | -2.281579876 | 0.093 | down |
| 2-Pyrrolidinone | 1.044942582 | 2.307690319 | 1.206449634 | 0.094 | up |
| 4-Methylvaleric Acid | 1.491225892 | 3.823011382 | 1.934709495 | 0.094 | up |
| Adipic Acid | 1.330241616 | 2.040773492 | 1.029116065 | 0.095 | up |
| N6-Acetyl-L-Lysine | 1.337038941 | 0.490485934 | -1.027716331 | 0.098 | down |
| Valeric Acid | 1.272569039 | 2.200400255 | 1.137765975 | 0.101 | up |
| A-Ketoglutaric Acid | 1.31867968 | 2.016597318 | 1.01192303 | 0.102 | up |
| Raffinose | 1.366417675 | 0.373728647 | -1.419936941 | 0.103 | down |
| Maltol | 1.162562379 | 2.84867956 | 1.510293346 | 0.112 | up |
| 2,4-Dihydroxypteridine | 1.612664112 | 0.330887358 | -1.595587924 | 0.114 | down |
| N'-Formylkynurenine | 1.378238654 | 0.295407613 | -1.759221089 | 0.115 | down |
| 4-Hydroxy-L-Glutamic Acid | 1.241214346 | 0.494478158 | -1.0160213 | 0.119 | down |
| Syringic Acid | 1.222716272 | 2.141191345 | 1.098413726 | 0.124 | up |
| Phenylalanine-Phenylalanine | 1.019580553 | 2.326730913 | 1.218304372 | 0.130 | up |
| Glycerol 3-phosphate | 1.245142293 | 0.335358088 | -1.576225698 | 0.133 | down |
| Caffeic Acid | 1.388341915 | 0.340019999 | -1.556308493 | 0.137 | down |
| Lysopc 18:1 | 1.288852926 | 0.479840445 | -1.05937333 | 0.137 | down |
| 1,5-Diaminopentane | 1.073747468 | 0.308367869 | -1.697275647 | 0.137 | down |
| Compounds | VIP | FC | Log_2_FC | p | Type |
| 2-Hydroxy-6-Aminopurine | 1.306756037 | 0.451741961 | -1.146429166 | 0.138 | down |
| 3-Hydroxyglutaric acid | 1.252100033 | 2.092051517 | 1.064918379 | 0.143 | up |
| δ-Valerolactam | 1.18118448 | 0.474916012 | -1.074255697 | 0.144 | down |
| 4-Hydroxybenzyl alcohol | 1.292969811 | 0.478539538 | -1.063289966 | 0.146 | down |
| 11,12-EET [(±)11,(12)-epoxy-5Z,8Z,14Z-eicosatrienoic acid] | 1.564744091 | 0.187572031 | -2.414483373 | 0.149 | down |
| 2-Phenylacetamide | 1.017832547 | 0.279050274 | -1.841403029 | 0.149 | down |
| Oxoadipic Acid | 1.262037722 | 2.593307736 | 1.374793414 | 0.157 | up |
| ADP-ribose | 1.476172184 | 9.41362153 | 3.234749851 | 0.158 | up |
| Cyclohexylamine | 1.22016817 | 0.360896107 | -1.470344515 | 0.161 | down |
| 15-oxoETE [15-oxo-5Z,8Z,11Z,13E-eicosatetraenoic acid] | 1.230605104 | 0.21257234 | -2.233974213 | 0.165 | down |
| L-Cystathionine | 1.100139481 | 0.169194376 | -2.563246484 | 0.172 | down |
| L-Pyroglutamic Acid | 1.334897726 | 0.386075532 | -1.37304497 | 0.177 | down |
| 1-(4-Methoxyphenyl)-2-propanone | 1.336047378 | 2.399646254 | 1.262821745 | 0.181 | up |
| DL-Carnitine | 1.616012452 | 0.198181971 | -2.335102373 | 0.181 | down |
| 3,5-Dimethoxy-4-Hydroxycinnamic Acid | 1.25088094 | 5.002518617 | 2.322654631 | 0.184 | up |
| L-Asparagine Anhydrous | 1.37706716 | 0.461826858 | -1.114576019 | 0.185 | down |
| 3-Aminoisobutanoic Acid | 1.302482886 | 0.151599077 | -2.721667126 | 0.188 | down |
| D-Melezitose | 1.153629645 | 0.337388229 | -1.567518453 | 0.190 | down |
| Glycyl-L-Proline | 1.235702507 | 0.455240713 | -1.135298508 | 0.191 | down |
| Malonicacid | 1.069196887 | 2.551427128 | 1.351304437 | 0.192 | up |
| N-acetylornithine | 1.227584861 | 0.449223825 | -1.154493652 | 0.192 | down |
| 3-Hydroxybutyrate | 1.050194159 | 2.522202457 | 1.334684085 | 0.193 | up |
| Hexadecanedioic acid | 1.257409213 | 0.329846874 | -1.600131665 | 0.197 | down |
| Butyl Acetate | 1.357249705 | 2.735894981 | 1.452012853 | 0.197 | up |
| Phosphocholine | 1.111151805 | 2.096136601 | 1.067732737 | 0.202 | up |
| Ergothioneine | 1.59611555 | 0.144476554 | -2.791092708 | 0.204 | down |
| 2,4-Dihydroxybenzoic Acid | 1.170037261 | 0.346271013 | -1.530026474 | 0.206 | down |
| Glycerophosphatidylcholine | 1.315772518 | 0.112509948 | -3.151875523 | 0.209 | down |
| N-Acetyl-L-Histidine | 1.206810871 | 0.271877237 | -1.878972728 | 0.209 | down |
| Sn-Glycero-3-Phosphocholine | 1.349875783 | 0.102655427 | -3.284118192 | 0.210 | down |
| 3-Amino-4-Hydroxybenzoic Acid | 1.048017193 | 0.471673417 | -1.084139799 | 0.211 | down |
| D-Arabitol | 1.281661691 | 0.303299056 | -1.721187088 | 0.213 | down |
| L-Arginine | 1.162813512 | 0.442440825 | -1.176443583 | 0.215 | down |
| Arachidic Acid(C20:0) | 1.195782492 | 0.258387384 | -1.952392463 | 0.219 | down |
| Lysopc 16:1 | 1.45944764 | 0.282999619 | -1.821127982 | 0.222 | down |
| N-Acetylphenylalanine | 1.07099133 | 0.48766544 | -1.036036359 | 0.222 | down |
| 5-HETrE [5S-hydroxy-6E,8Z,11Z-eicosatrienoic acid] | 1.168367447 | 0.362119297 | -1.465463035 | 0.234 | down |
| Acetyl-L-carnitine | 1.208584649 | 0.159263423 | -2.650513126 | 0.273 | down |
| Indolelactic acid | 1.042307144 | 2.309549789 | 1.207611648 | 0.274 | up |
| L-Carnitine | 1.01074291 | 0.326769122 | -1.613656433 | 0.302 | down |

**Table S2.** Summary of the distinct metabolites between CG-0W and CG-8W

| Compounds | VIP | FC | Log_2_FC | P | Type |
| --- | --- | --- | --- | --- | --- |
| 2'-Hydroxy-5'-methylacetophenone | 2.1994 | 3.5305797 | 1.819905 | **0.000** | up |
| 3-Ureidopropionate | 2.193494 | 2.7288934 | 1.448316 | **0.000** | up |
| Enterodiol | 2.245728 | 0.0630672 | -3.98697 | **0.000** | down |
| 7,8-dihydro-L-Biopterin | 2.048518 | 0.4669986 | -1.09851 | **0.001** | down |
| 2,5-Furandicarboxylicacid | 2.074436 | 2.9454299 | 1.558478 | **0.001** | up |
| Decanal | 2.086261 | 3.0592098 | 1.613159 | **0.001** | up |
| 1,7-Dimethylxanthine | 1.99879 | 3.2544425 | 1.70241 | **0.002** | up |
| Cis-11,14-Eicosadienoic Acid (C20:2) | 1.981533 | 0.2281513 | -2.13194 | **0.006** | down |
| L-Lysine | 1.890303 | 0.4930804 | -1.02011 | **0.006** | down |
| Pseudouridine | 1.884796 | 2.8268358 | 1.499188 | **0.009** | up |
| 3-(4-Hydroxyphenyl)-Propionic Acid | 1.363984 | 8.7129713 | 3.123165 | **0.010** | up |
| 2,4-Dihydroxybenzoic Acid | 1.804561 | 0.2349482 | -2.08959 | **0.015** | down |
| Lumichrome | 2.094185 | 6.9265337 | 2.792134 | **0.016** | up |
| 3,4,5-Trimethoxybenzoic Acid | 1.658624 | 2.3189168 | 1.213451 | **0.017** | up |
| Imidazoleacetic acid | 1.960164 | 5.3676006 | 2.424277 | **0.017** | up |
| 15-Deoxy-δ-12,14-PGJ2 | 1.721341 | 0.3263494 | -1.61551 | **0.018** | down |
| Ergothioneine | 2.025977 | 0.2509096 | -1.99476 | **0.019** | down |
| LysoPE(16:1(9Z)/0:0) | 1.928494 | 4.4558988 | 2.155716 | **0.020** | up |
| PGF3α [9α,11α,15S-trihydroxy-prosta-5Z,13E,17Z-trien-1-oic acid] | 1.75581 | 0.3913327 | -1.35353 | **0.021** | down |
| P-Aminobenzoate | 1.726821 | 11.133341 | 3.476815 | **0.022** | up |
| Choline chloride | 1.855387 | 0.255111 | -1.9708 | **0.023** | down |
| 4-Hydroxy-L-Glutamic Acid | 1.515736 | 0.3822304 | -1.38749 | **0.023** | down |
| Valeric Acid | 1.714399 | 2.0744121 | 1.052703 | **0.024** | up |
| Homovanillic Acid | 1.736928 | 11.303865 | 3.498744 | **0.028** | up |
| Propylpropionate | 1.431955 | 0.4508364 | -1.14932 | **0.028** | down |
| 2,6-Diaminooimelic Acid | 1.75313 | 0.4895701 | -1.03041 | **0.028** | down |
| 2-Hydroxy-6-Aminopurine | 1.756724 | 0.3112094 | -1.68404 | **0.028** | down |
| Indole-3-Pyruvic Acid | 2.008987 | 4.4938066 | 2.167938 | **0.028** | up |
| 4-Hydroxyretinoic Acid | 1.656536 | 0.3135303 | -1.67332 | **0.033** | down |
| Carbamoyl phosphate | 1.663607 | 2.6634717 | 1.413308 | **0.034** | up |
| Benzoic Acid | 1.854389 | 6.4747104 | 2.694816 | **0.034** | up |
| 3,5-Dimethoxy-4-Hydroxycinnamic Acid | 1.72936 | 5.4816655 | 2.454614 | **0.040** | up |
| 5,6-DiHETrE [(±)5,6-dihydroxy-8Z,11Z,14Z-eicosatrienoic acid] | 1.807444 | 0.3286898 | -1.6052 | **0.042** | down |
| (±)12-HETE [(±)12-hydroxy-5Z,8Z,10E,14Z-eicosatetraenoic acid] | 1.589524 | 0.3340938 | -1.58168 | **0.042** | down |
| Choline | 1.7367 | 0.3183181 | -1.65146 | **0.045** | down |
| Methylmalonic Acid | 1.64058 | 4.7429075 | 2.245772 | **0.045** | up |
| Arachidic Acid(C20:0) | 1.733474 | 0.1434626 | -2.80125 | **0.048** | down |
| 3-(3-Hydroxyphenyl)Propionate Acid | 1.339153 | 0.1147992 | -3.12282 | 0.050 | down |
| D-Sorbitol | 1.83299 | 0.1711735 | -2.54647 | 0.050 | down |
| L-3-Phenyllactic Acid | 1.425526 | 0.1777435 | -2.49213 | 0.050 | down |
| PGF2α [9α,11α,15S-trihydroxy-prosta-5Z,13E-dien-1-oic acid] | 1.615192 | 0.4030028 | -1.31114 | 0.051 | down |
| Trimethylamine N-Oxide | 1.483268 | 4.263585 | 2.092067 | 0.052 | up |
| Compounds | VIP | FC | Log_2_FC | P | Type |
| P-Coumaraldehyde | 1.325786 | 0.1136823 | -3.13692 | 0.052 | down |
| L-Carnosine | 1.792572 | 0.3401669 | -1.55569 | 0.053 | down |
| 3-Amino-4-Hydroxybenzoic Acid | 1.781388 | 0.2794699 | -1.83923 | 0.053 | down |
| Glycylphenylalanine | 1.500738 | 0.4479727 | -1.15852 | 0.060 | down |
| 2-(Dimethylamino)Guanosine | 1.584953 | 0.3668742 | -1.44664 | 0.065 | down |
| N'-Formylkynurenine | 1.590366 | 0.3459624 | -1.53131 | 0.066 | down |
| O-Succinyl-L-Homoserine | 1.431329 | 0.4249954 | -1.23448 | 0.067 | down |
| O-Phosphorylethanolamine | 1.431345 | 2.25429 | 1.172673 | 0.069 | up |
| 1,3-Diaminopropane | 1.740178 | 3.9429967 | 1.979293 | 0.070 | up |
| Syringic Acid | 1.413161 | 3.4492819 | 1.786296 | 0.070 | up |
| 1-(4-Methoxyphenyl)-2-propanone | 1.54755 | 0.4659771 | -1.10167 | 0.073 | down |
| 5-Hydroxyhexanoic Acid | 1.115327 | 2.2132137 | 1.146143 | 0.076 | up |
| 2,5-Dihydroxy Benzoic Acid | 1.757418 | 0.2386868 | -2.06681 | 0.076 | down |
| Succinic Acid | 1.481603 | 3.9590382 | 1.98515 | 0.081 | up |
| Diethyl malonate | 1.056914 | 0.3133391 | -1.6742 | 0.082 | down |
| Methyl Indole-3-Acetate | 1.429732 | 2.633293 | 1.396868 | 0.085 | up |
| Acetyl-L-carnitine | 1.163672 | 2.0256121 | 1.018358 | 0.086 | up |
| D-Glucoronic Acid | 1.114916 | 0.2962365 | -1.75518 | 0.087 | down |
| Acetyl Tryptophan | 1.107585 | 0.3074658 | -1.7015 | 0.090 | down |
| 3-Hydroxy-3-Methyl Butyric Acid | 1.615674 | 2.7663303 | 1.467973 | 0.098 | up |
| 1,5-Anhydro-D-Glucitol | 1.452318 | 0.4607427 | -1.11797 | 0.099 | down |
| Pyrrole-2-Carboxylic Acid | 1.725546 | 8.107241 | 3.019211 | 0.100 | up |
| Indole-3-Carboxaldehyde | 1.204797 | 2.4399744 | 1.286866 | 0.105 | up |
| Lactulose | 1.270858 | 0.4056434 | -1.30172 | 0.106 | down |
| N-Glycyl-L-Leucine | 1.39316 | 0.4209094 | -1.24842 | 0.111 | down |
| Argininosuccinic acid | 1.162617 | 0.1648904 | -2.60042 | 0.113 | down |
| D-Glucuronolactone | 1.036587 | 0.326275 | -1.61584 | 0.113 | down |
| Dulcitol | 1.39945 | 0.3452096 | -1.53446 | 0.113 | down |
| Cytidine | 1.329813 | 0.436898 | -1.19463 | 0.114 | down |
| Nicotinamide | 1.508238 | 6.4135627 | 2.681126 | 0.120 | up |
| Vanillin | 1.113822 | 0.4728002 | -1.0807 | 0.138 | down |
| 3-Methoxytyramine | 1.116513 | 2.3228199 | 1.215877 | 0.141 | up |
| Marmesin | 1.046391 | 0.4802738 | -1.05807 | 0.143 | down |
| Phosphocholine | 1.376278 | 0.4748695 | -1.0744 | 0.150 | down |
| 3-Hydroxymandelate | 1.280954 | 0.3891554 | -1.36158 | 0.150 | down |
| D-Glucose 6-Phosphate | 1.050228 | 0.2955024 | -1.75876 | 0.163 | down |
| Lysopc 20:1 | 1.485282 | 0.1044457 | -3.25918 | 0.167 | down |
| N-lactoyl-phenylalanine | 1.416906 | 0.2926767 | -1.77262 | 0.167 | down |
| alpha-Cadinene | 1.719504 | 0.1280137 | -2.96563 | 0.169 | down |
| 9-HOTrE [9S-hydroxy-10E,12Z,15Z-octadecatrienoic acid] | 1.80167 | 0.0927313 | -3.4308 | 0.169 | down |
| beta-Cubebene | 1.710662 | 0.1280356 | -2.96538 | 0.170 | down |
| Caffeic Acid | 1.396387 | 0.2143013 | -2.22229 | 0.171 | down |
| N-Isovaleroylglycine | 1.669185 | 8.496938 | 3.086943 | 0.179 | up |
| Compounds | VIP | FC | Log_2_FC | P | Type |
| 7-ketodeoxycholic acid | 1.155638 | 0.4127616 | -1.27662 | 0.180 | down |
| D-Xylulose 5-phosphate | 1.293572 | 0.2299557 | -2.12057 | 0.180 | down |
| Hyodeoxycholic Acid | 1.004424 | 2.0581692 | 1.041362 | 0.182 | up |
| Lysopc 14:0 | 1.374669 | 0.1407543 | -2.82875 | 0.183 | down |
| 3-(3-Hydroxyphenyl)-3-hydroxypropanoic acid | 1.226354 | 0.0802639 | -3.63911 | 0.184 | down |
| D-Fructose 6-Phosphate-Disodium Salt | 1.030179 | 0.3490583 | -1.51846 | 0.189 | down |
| Punicic Acid | 1.259652 | 0.2681458 | -1.89891 | 0.191 | down |
| Guanosine Monophosphate | 1.276293 | 0.1303076 | -2.94001 | 0.196 | down |
| 11,12-EET [(±)11,(12)-epoxy-5Z,8Z,14Z-eicosatrienoic acid] | 1.438963 | 0.1966812 | -2.34607 | 0.202 | down |
| Indole-3-Ethanol | 1.278991 | 0.4640927 | -1.10752 | 0.203 | down |
| (±)5-HETE [(±)5-hydroxy-6E,8Z,11Z,14Z-eicosatetraenoic acid] | 1.327775 | 0.2662164 | -1.90933 | 0.205 | down |
| 5'-Deoxy-5'-(Methylthio) Adenosine | 1.024723 | 2.089217 | 1.062962 | 0.209 | up |
| O-Phospho-L-Serine | 1.117825 | 2.0734216 | 1.052014 | 0.211 | up |
| Lysopc 16:0 | 1.146211 | 0.4147601 | -1.26965 | 0.219 | down |
| 9,10-DiHOME [(±)9,10-dihydroxy-12Z-octadecenoic acid] | 1.080769 | 0.2461835 | -2.02219 | 0.220 | down |
| Ribulose-5-Phosphate | 1.126779 | 0.2847955 | -1.812 | 0.221 | down |
| Lysopc 18:3 | 1.092476 | 0.3624744 | -1.46405 | 0.221 | down |
| m-Coumaric acid | 1.254249 | 0.2119808 | -2.23799 | 0.230 | down |
| trans-3-Indoleacrylic acid | 1.26184 | 0.323492 | -1.6282 | 0.234 | down |
| Uric Acid | 1.018001 | 0.4290141 | -1.2209 | 0.238 | down |
| L-Tryptophan | 1.1616 | 0.3521025 | -1.50593 | 0.238 | down |
| Tryptamine | 1.140074 | 0.1772941 | -2.49578 | 0.239 | down |
| N-Acetylaspartylglutamic acid | 1.066 | 2.8926216 | 1.532378 | 0.239 | up |
| N-Acetyl-L-alanine | 1.194346 | 5.6188726 | 2.490281 | 0.247 | up |
| Methoxyindoleacetic Acid | 1.153694 | 0.3511965 | -1.50965 | 0.248 | down |
| Lysopc 17:0 | 1.117234 | 0.4259102 | -1.23138 | 0.257 | down |
| 13-HOTrE [13S-hydroxy-9Z,11E,15Z-octadecatrienoic acid] | 1.520829 | 0.2888595 | -1.79156 | 0.276 | down |
| N-Acetylglucosamine 1-Phosphate | 1.222057 | 0.3977424 | -1.33009 | 0.308 | down |
| 3-Aminoisobutanoic Acid | 1.132355 | 0.4239581 | -1.23801 | 0.334 | down |

**Table S3.** Summary of the distinct metabolites between OG-8W and CG-8W

| Compounds | VIP | FC | Log2FC | P | Type |
| --- | --- | --- | --- | --- | --- |
| Maleic Acid | 2.06534284 | 2.178903669 | 1.12360242 | **0.000** | up |
| 2,6-Dihydroxypurine | 2.008614213 | 2.352945932 | 1.23446817 | **0.000** | up |
| L-Methionine | 1.998050618 | 2.316721177 | 1.21208442 | **0.001** | up |
| Glycine | 2.0029185 | 2.299975393 | 1.20161843 | **0.001** | up |
| 5-Aminolevulinate | 1.974081437 | 2.763384552 | 1.46643634 | **0.001** | up |
| N,N-Dimethylglycine | 1.828046798 | 2.338831289 | 1.2257878 | **0.001** | up |
| Pyrrolidine | 1.924127651 | 2.300172878 | 1.2017423 | **0.001** | up |
| 6-Aminocaproic Acid | 1.884761683 | 2.450604471 | 1.29313765 | **0.002** | up |
| N-methylalanine | 1.835313352 | 2.222890442 | 1.15243684 | **0.002** | up |
| DL-Carnitine | 1.881226882 | 2.682898198 | 1.42379231 | **0.002** | up |
| 5-Aminovaleric Acid | 1.527296067 | 3.095383339 | 1.63011809 | **0.002** | up |
| Guanidineacetic Acid | 1.517570584 | 3.108621378 | 1.63627491 | **0.002** | up |
| Piperidine | 1.866194097 | 2.450105141 | 1.29284366 | **0.002** | up |
| 2'-Deoxyuridine | 1.722599448 | 2.391499236 | 1.25791533 | **0.003** | up |
| Indoleacetaldehyde | 1.811425576 | 2.540255875 | 1.34497382 | **0.003** | up |
| 6-Phosphogluconic Acid Trisodium Salt | 1.725732334 | 2.287477634 | 1.19375764 | **0.004** | up |
| Indole-3-Acetic Acid | 1.781074553 | 2.381502232 | 1.2518719 | **0.004** | up |
| Trans-4-Hydroxy-L-Proline | 1.883286432 | 3.256324644 | 1.70324454 | **0.004** | up |
| 5-Hydroxy-L-Tryptophan | 1.791258843 | 2.570887329 | 1.36226638 | **0.005** | up |
| L-Isoleucine | 1.881774717 | 3.194806203 | 1.67572842 | **0.005** | up |
| Cis-11,14,17-Eicosatrienoic Acid (C20:3) | 1.210682399 | 0.489488797 | -1.03065225 | **0.006** | down |
| L-Threonine | 1.761318331 | 2.161518631 | 1.11204527 | **0.006** | up |
| Indole | 1.803970756 | 2.486483528 | 1.31410687 | **0.007** | up |
| Urocanic Acid | 1.933979314 | 0.243740322 | -2.03658316 | **0.008** | down |
| Aspartame | 1.755085145 | 3.798869183 | 1.92557003 | **0.008** | up |
| L-Serine | 1.821635044 | 2.262644171 | 1.17800972 | **0.008** | up |
| L-Thyroxine | 1.823841038 | 5.947453866 | 2.57227218 | **0.009** | up |
| Cis-3-Hexenylacetate | 1.782336063 | 2.32791294 | 1.2190371 | **0.009** | up |
| 2-Nonanone | 1.793722115 | 2.387061281 | 1.2552356 | **0.009** | up |
| 3-Hydroxypicolinic acid | 1.911415566 | 0.250706204 | -1.9959304 | **0.010** | down |
| L-Alanine | 1.847543072 | 2.678264697 | 1.42129855 | **0.011** | up |
| L-Norleucine | 1.641975648 | 2.040958823 | 1.02924708 | **0.011** | up |
| N-Acetylmannosamine | 1.471753697 | 4.149672888 | 2.05299762 | **0.014** | up |
| Indole-3-Pyruvic Acid | 2.103794068 | 9.037253608 | 3.17588441 | **0.015** | up |
| 1,7-Dimethylxanthine | 1.562299495 | 2.115437439 | 1.08095602 | **0.017** | up |
| 2-Methylsuccinic Acid | 1.754263301 | 0.471279287 | -1.08534582 | **0.020** | down |
| Glutaric Acid | 1.747787323 | 0.468464078 | -1.09398967 | **0.020** | down |
| Deoxycorticosterone Acetate | 1.541297044 | 3.355962653 | 1.74672666 | **0.020** | up |
| L-Tryptophan | 1.713027753 | 4.295113745 | 2.10269634 | **0.020** | up |
| Cis-11,14-Eicosadienoic Acid (C20:2) | 1.695603755 | 0.232479533 | -2.10482438 | **0.020** | down |
| N-Acetyl-D-Glucosamine | 1.451215133 | 2.994130053 | 1.58213689 | **0.021** | up |
| Glycyl-L-Proline | 1.538716496 | 2.143614147 | 1.10004524 | **0.025** | up |
| Compounds | VIP | FC | Log2FC | P | Type |
| 10-Undecenoic Acid | 1.623167255 | 0.478871651 | -1.06228906 | **0.027** | down |
| O-Desmethylnaproxen | 1.790922144 | 0.330296374 | -1.59816696 | **0.028** | down |
| Indole-3-Carbinol | 1.484673943 | 3.335804154 | 1.73803459 | **0.028** | up |
| δ-Valerolactam | 1.420106435 | 2.916136032 | 1.54405802 | **0.029** | up |
| D-Sorbitol | 1.94694455 | 0.109900933 | -3.18572446 | **0.030** | down |
| 3,3',5-Triiodo-L-Thyronine | 1.535715594 | 3.446139383 | 1.78498105 | **0.030** | up |
| 2-nonanol | 1.027323705 | 2.643060104 | 1.40220923 | **0.030** | up |
| N-Methylephedrine | 1.612628782 | 0.371891208 | -1.42704745 | **0.030** | down |
| Methoxyindoleacetic Acid | 1.08101945 | 3.711481173 | 1.89199505 | **0.031** | up |
| Cyclohexylamine | 1.413172992 | 4.626642866 | 2.20996574 | **0.033** | up |
| Carbamoyl phosphate | 1.584616054 | 2.651564669 | 1.40684393 | **0.033** | up |
| Pseudouridine | 1.513342569 | 2.058053572 | 1.04128054 | **0.037** | up |
| Isoquinoline | 1.467674059 | 3.322925747 | 1.73245406 | **0.038** | up |
| N-Alpha-acetyllysine | 1.559329426 | 2.199377871 | 1.13709549 | **0.038** | up |
| Methyl Indole-3-Acetate | 1.542331684 | 2.744114259 | 1.45634055 | **0.039** | up |
| Glycocholic Acid | 1.063388528 | 4.509153324 | 2.17285657 | **0.043** | up |
| Indole-3-Carboxaldehyde | 1.389625748 | 3.350512865 | 1.74438195 | **0.045** | up |
| 2-Hydroxyisocaproic Acid | 1.609191877 | 3.24678021 | 1.69900973 | **0.047** | up |
| Dulcitol | 1.635630365 | 0.237854993 | -2.07184578 | **0.048** | down |
| 2,4-Dihydroxypteridine | 1.364169617 | 2.596356628 | 1.37648856 | 0.051 | up |
| Thiamine | 1.475730329 | 2.933350359 | 1.5525494 | 0.052 | up |
| Glycoursodeoxycholic Acid | 1.542455791 | 2.767680535 | 1.46867743 | 0.052 | up |
| 3-(4-Hydroxyphenyl)-Propionic Acid | 1.331282866 | 2.128973452 | 1.09015796 | 0.056 | up |
| O-Phosphorylethanolamine | 1.423720709 | 2.295043528 | 1.19852152 | 0.063 | up |
| N-Acetylaspartylglutamic acid | 1.745407312 | 5.166134491 | 2.3690852 | 0.063 | up |
| Marmesin | 1.32502327 | 0.459180855 | -1.1228656 | 0.064 | down |
| Lumichrome | 1.592914259 | 2.95203049 | 1.56170762 | 0.068 | up |
| trans-3-Indoleacrylic acid | 1.28508205 | 2.51047219 | 1.32795874 | 0.068 | up |
| Indolelactic acid | 1.107659953 | 0.321927579 | -1.63519192 | 0.068 | down |
| Vanillic Acid | 1.020981374 | 2.536495573 | 1.34283664 | 0.069 | up |
| 3-Hydroxyglutaric acid | 1.602100279 | 0.376092036 | -1.41084234 | 0.071 | down |
| N'-Formylkynurenine | 1.490258637 | 2.276847146 | 1.18703744 | 0.074 | up |
| 2-Aminoethanesulfinic Acid | 1.490897613 | 0.447357819 | -1.16049886 | 0.078 | down |
| 3-Methoxytyramine | 1.34124858 | 3.054786996 | 1.61107179 | 0.078 | up |
| Dethiobiotin | 1.432019717 | 0.250840574 | -1.99515737 | 0.079 | down |
| 2,6-Dimethylpyridine | 1.516446388 | 0.457060273 | -1.12954367 | 0.080 | down |
| L-Aspartic Acid | 1.344438958 | 4.810097343 | 2.26606609 | 0.081 | up |
| P–Hydroxyphenyl Acetic Acid | 1.446780615 | 4.048252889 | 2.01729942 | 0.082 | up |
| Hyodeoxycholic Acid | 1.403018137 | 3.359040706 | 1.74804928 | 0.085 | up |
| Mandelic Acid | 1.455354176 | 4.11265387 | 2.04006966 | 0.086 | up |
| 3-Chloro-L-Tyrosine | 1.250790041 | 5.013704159 | 2.32587687 | 0.087 | up |
| Pantothenol | 1.670306242 | 0.14160511 | -2.82005477 | 0.093 | down |
| Lactulose | 1.380410927 | 0.465550264 | -1.10299115 | 0.094 | down |
| Compounds | VIP | FC | Log2FC | P | Type |
| Methylmalonic Acid | 1.270947558 | 2.758022924 | 1.46363445 | 0.098 | up |
| L-Arabitol | 1.650007451 | 2.451465647 | 1.29364455 | 0.105 | up |
| Homovanillic Acid | 1.100334301 | 3.070032812 | 1.61825408 | 0.108 | up |
| 6-Hydroxynicotinic Acid | 1.364093392 | 2.461487558 | 1.29953045 | 0.110 | up |
| 3-N-Methyl-L-Histidine | 1.425475648 | 2.318404635 | 1.21313238 | 0.113 | up |
| P-Aminobenzoate | 1.015414135 | 2.782227474 | 1.47624038 | 0.114 | up |
| Dodecanedioic Aicd | 1.371861199 | 0.432417609 | -1.20950282 | 0.117 | down |
| Butyl Acetate | 1.602485369 | 0.226842083 | -2.14023978 | 0.119 | down |
| 2,3-Dinor-TXB2 | 1.016733999 | 2.953262562 | 1.56230963 | 0.119 | up |
| Oxoadipic Acid | 1.361008429 | 0.315551662 | -1.66405187 | 0.121 | down |
| N6-Acetyl-L-Lysine | 1.151199161 | 2.065933752 | 1.04679399 | 0.121 | up |
| Methionine Sulfoxide | 1.316719621 | 2.042653456 | 1.03044447 | 0.131 | up |
| Kinic Acid | 1.525232503 | 0.335436154 | -1.5758899 | 0.131 | down |
| 3-(3-Hydroxyphenyl)-3-hydroxypropanoic acid | 1.447026842 | 0.10683241 | -3.2265787 | 0.136 | down |
| 1-(4-Methoxyphenyl)-2-propanone | 1.406793193 | 0.381598495 | -1.38987261 | 0.137 | down |
| Enterodiol | 1.78592763 | 0.083965727 | -3.57405562 | 0.141 | down |
| Phosphocholine | 1.37915486 | 0.35600896 | -1.49001454 | 0.141 | down |
| Orotic Acid | 1.216956901 | 2.528033399 | 1.33801552 | 0.143 | up |
| Succinic Acid | 1.152511511 | 2.598834466 | 1.37786474 | 0.145 | up |
| 4-Hydroxybenzoic Acid | 1.239950096 | 2.143910413 | 1.10024462 | 0.145 | up |
| D-Mannitol | 1.421810073 | 0.376102639 | -1.41080166 | 0.146 | down |
| 2-Methoxybenzoic Acid | 1.180461871 | 4.327124598 | 2.11340866 | 0.151 | up |
| 3-Hydroxyanthranilic Acid | 1.093241601 | 3.88719643 | 1.95873001 | 0.153 | up |
| Methyl Nicotinate | 1.027141049 | 2.688647472 | 1.42688061 | 0.153 | up |
| Β-Nicotinamide Mononucleotide | 1.327415627 | 0.260410342 | -1.94114135 | 0.156 | down |
| O-Phospho-L-Serine | 1.24851645 | 2.40244478 | 1.26450327 | 0.164 | up |
| Thioguanine | 1.064295296 | 2.07798398 | 1.05518453 | 0.169 | up |
| Citramalic Acid | 1.344415892 | 0.364877271 | -1.45451681 | 0.174 | down |
| Hydroquinone | 1.416812075 | 4.23906428 | 2.08374584 | 0.180 | up |
| Anthranilic acid | 1.026970849 | 3.017949748 | 1.59356878 | 0.187 | up |
| 3-Hydroxybutyrate | 1.175790642 | 0.36993525 | -1.43465532 | 0.192 | down |
| Malonicacid | 1.165556916 | 0.376334583 | -1.40991222 | 0.199 | down |
| 12,13-EpOME [(±)12(13)epoxy-9Z-octadecenoic acid] | 1.009342787 | 0.371168911 | -1.42985222 | 0.203 | down |
| 3-Aminoisobutanoic Acid | 1.14167121 | 3.309381061 | 1.72656142 | 0.209 | up |
| O-Succinyl-L-Homoserine | 1.021737152 | 0.48643002 | -1.03969583 | 0.213 | down |
| L-Cystathionine | 1.065389984 | 3.261842103 | 1.70568695 | 0.223 | up |
| 9-HOTrE [9S-hydroxy-10E,12Z,15Z-octadecatrienoic acid] | 1.112926483 | 0.112229125 | -3.15548097 | 0.230 | down |
| Lysopc 18:1 | 1.025374182 | 2.102924986 | 1.07239739 | 0.237 | up |
| 2-Aminoethanesulfonic Acid | 1.278741748 | 2.170003821 | 1.11769758 | 0.263 | up |
| Punicic Acid | 1.050600706 | 0.217867095 | -2.19847977 | 0.271 | down |
| 9,10-EpOME [(±)9,10-epoxy-12Z-octadecenoic acid] | 1.061524506 | 0.467608897 | -1.09662571 | 0.286 | down |
| 2-Phenylacetamide | 1.020976147 | 6.20004992 | 2.63227983 | 0.318 | up |
| Lysopc 16:1 | 1.07392961 | 3.344068776 | 1.74160452 | 0.354 | up |

**Table** **S4**: Quantitative results of SCFAs in each week-group (ug/mg)

| Sample_ID | Group | AA | BA | HA | IBA | IVA | PA | VA |
| --- | --- | --- | --- | --- | --- | --- | --- | --- |
| CG-0W-1 | CG-0W | 1.050 | 0.238 | N/A | 0.012 | 0.011 | 0.641 | 0.020 |
| CG-0W-2 |  | 5.000 | 1.500 | 0.246 | 0.044 | 0.043 | 0.746 | 0.265 |
| CG-0W-3 |  | 4.100 | 0.472 | 0.168 | 0.040 | 0.037 | 0.818 | 0.091 |
| CG-0W-4 |  | 2.350 | 0.288 | 0.045 | 0.038 | 0.029 | 0.531 | 0.073 |
| CG-0W-5 |  | 5.430 | 0.488 | 0.023 | 0.025 | 0.029 | 1.400 | 0.105 |
| CG-4W-1 | CG-4W | 4.740 | 0.448 | 0.019 | 0.064 | 0.110 | 1.820 | 0.168 |
| CG-4W-2 |  | 3.410 | 0.520 | 0.010 | 0.063 | 0.053 | 1.460 | 0.141 |
| CG-4W-3 |  | 4.100 | 0.094 | 0.132 | 0.349 | 0.390 | 1.570 | 0.175 |
| CG-4W-4 |  | 2.090 | 0.402 | 0.060 | 0.049 | 0.051 | 0.741 | 0.155 |
| CG-4W-5 |  | 3.510 | 0.616 | 0.060 | 0.116 | 0.102 | 1.650 | 0.212 |
| CG-8W-1 | CG-8W | 4.720 | 2.040 | 0.187 | 0.037 | 0.043 | 1.640 | 0.433 |
| CG-8W-2 |  | 2.180 | 0.555 | 0.171 | 0.077 | 0.096 | 0.992 | 0.238 |
| CG-8W-3 |  | 1.540 | 0.113 | 0.079 | 0.067 | 0.092 | 0.419 | 0.057 |
| CG-8W-4 |  | 1.690 | 0.308 | 0.066 | 0.061 | 0.068 | 0.980 | 0.225 |
| CG-8W-5 |  | 1.060 | 0.132 | 0.014 | 0.023 | 0.038 | 0.436 | 0.059 |
| OG-0W-1 | OG-0W | 2.910 | 0.414 | 0.048 | 0.040 | 0.038 | 0.517 | 0.090 |
| OG-0W-2 |  | 1.810 | 0.064 | 0.001 | 0.024 | 0.019 | 0.385 | 0.023 |
| OG-0W-3 |  | 2.230 | 0.182 | 0.057 | 0.041 | 0.039 | 0.832 | 0.098 |
| OG-0W-4 |  | 1.870 | 0.683 | 0.092 | 0.096 | 0.105 | 0.959 | 0.353 |
| OG-0W-5 |  | 3.270 | 1.290 | 0.004 | 0.017 | 0.017 | 0.896 | 0.094 |
| OG-4W-1 | OG-4W | 3.040 | 1.220 | 0.068 | 0.003 | 0.004 | 0.338 | 0.077 |
| OG-4W-2 |  | 0.238 | N/A | N/A | 0.005 | 0.000 | 0.037 | 0.000 |
| OG-4W-3 |  | 4.970 | 0.520 | 0.089 | 0.104 | 0.090 | 1.490 | 0.209 |
| OG-4W-4 |  | 1.410 | 0.387 | 0.007 | 0.078 | 0.123 | 1.020 | 0.152 |
| OG-4W-5 |  | 2.220 | 0.448 | 0.110 | 0.072 | 0.065 | 1.550 | 0.423 |
| OG-8W-1 | OG-8W | 5.580 | 2.460 | 0.381 | 0.044 | 0.048 | 1.030 | 0.518 |
| OG-8W-2 |  | 1.490 | 0.051 | 0.003 | 0.114 | 0.123 | 0.849 | 0.058 |
| OG-8W-3 |  | 3.520 | 0.495 | 0.102 | 0.065 | 0.070 | 1.650 | 0.334 |
| OG-8W-4 |  | 5.050 | 2.000 | 0.391 | 0.084 | 0.074 | 1.390 | 0.729 |
| OG-8W-5 |  | 2.950 | 1.120 | 0.033 | 0.027 | 0.028 | 2.400 | 0.748 |

*Notes:* N/A indicates that this substance has not been detected in this project, possibly because the content of this substance in the sample is lower than the detection limit of the instrument or the sample does not contain this substance; AA, Acetic acid; PA, Propionic acid; IBA, Isobutyric acid; BA, Butyric acid; IVA, Isovaleric acid; VA, Valeric acid; HA, Caproic acid.

**Supplementary Figure**


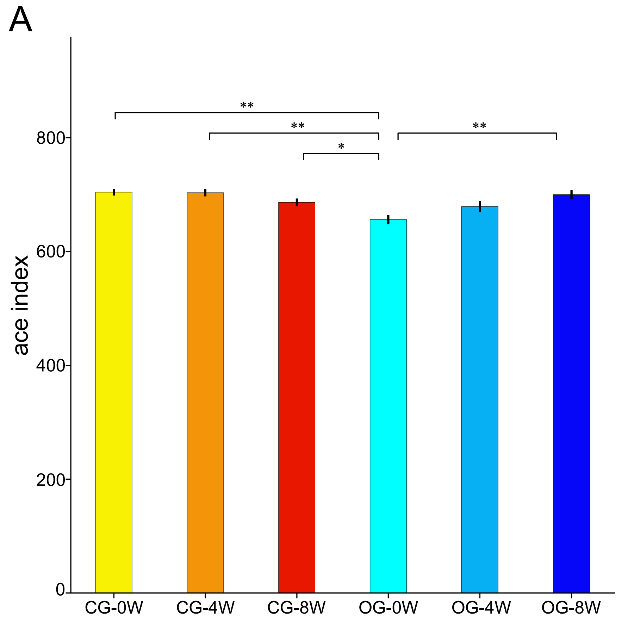

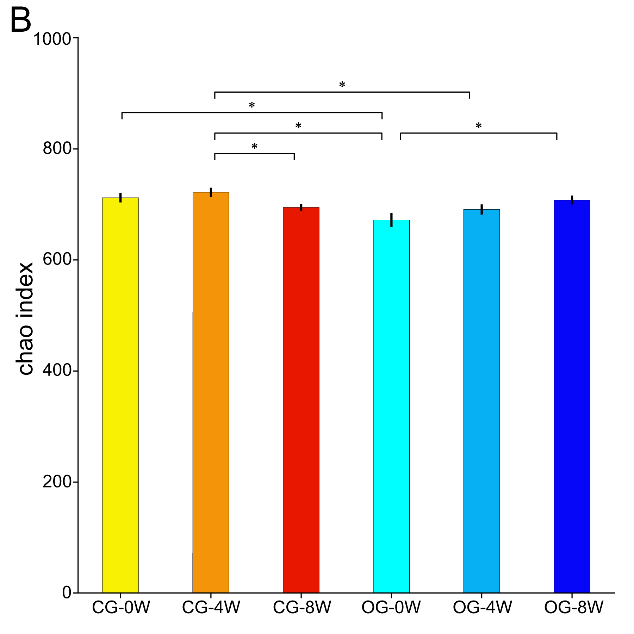


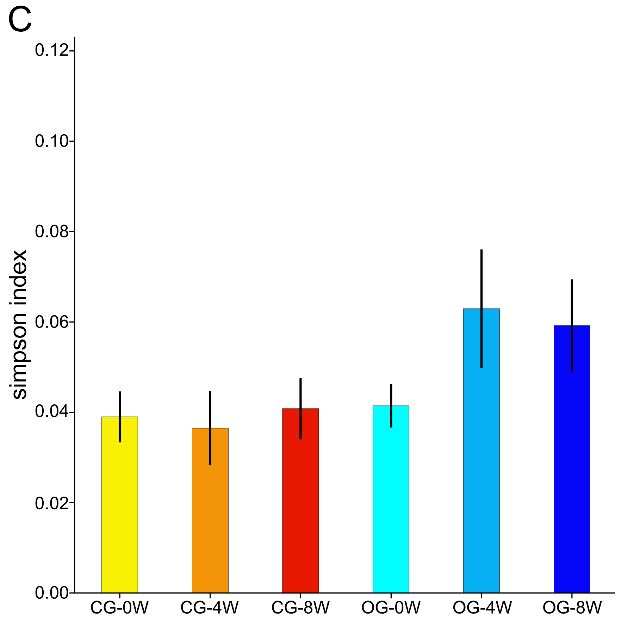

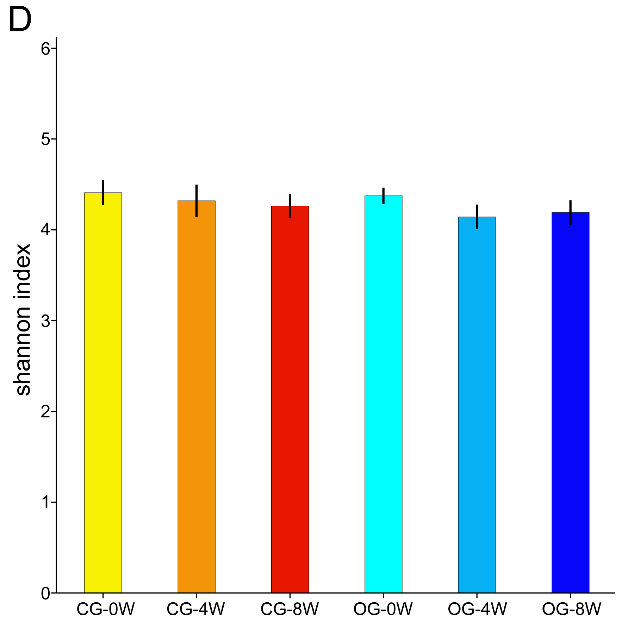


**Figure S1.** Alpha diversity index difference analysis of Bengal slow lorises among week-groups. (**a)** ace index. (**b)** chao index. (**c)** simpson index. (**d)** shannon index. Significant differences were marked as “*” (0.01 < P < 0.05) and “**” (P < 0.01).


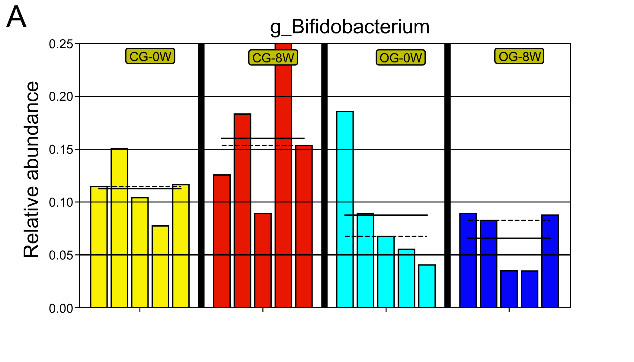

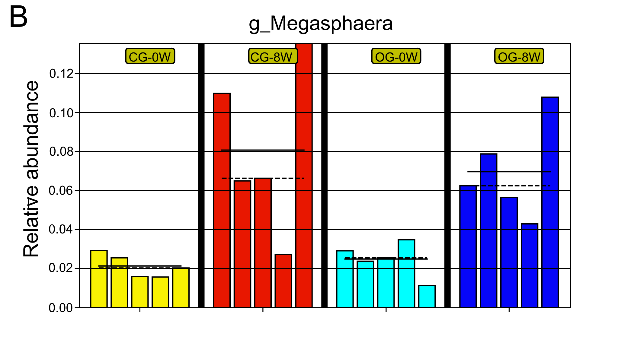

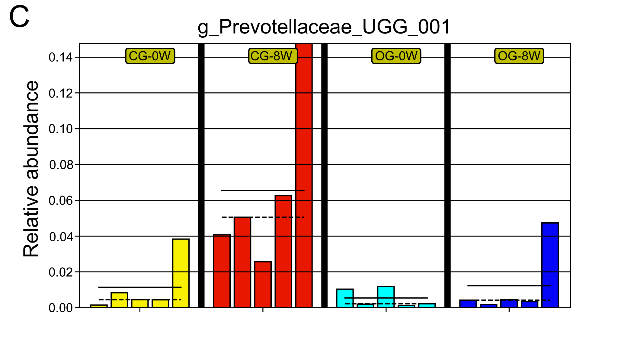

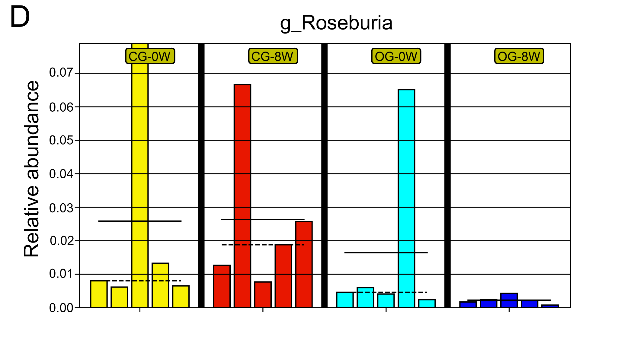

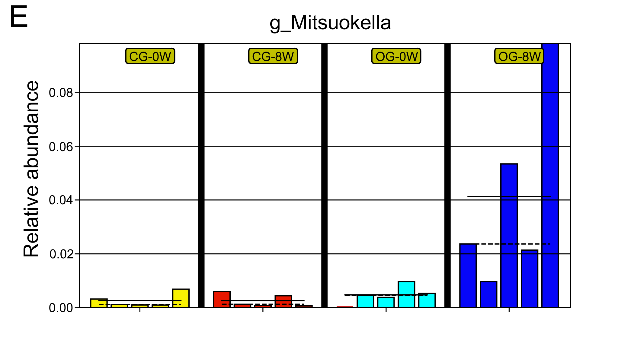

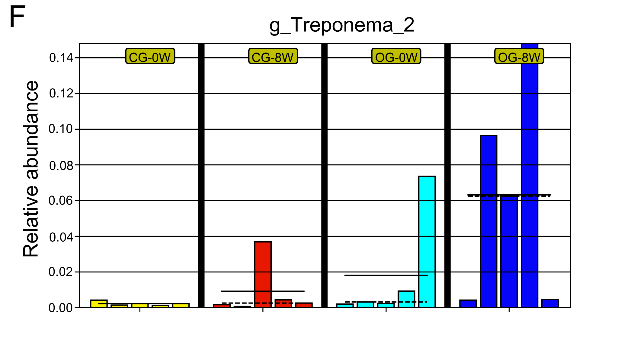

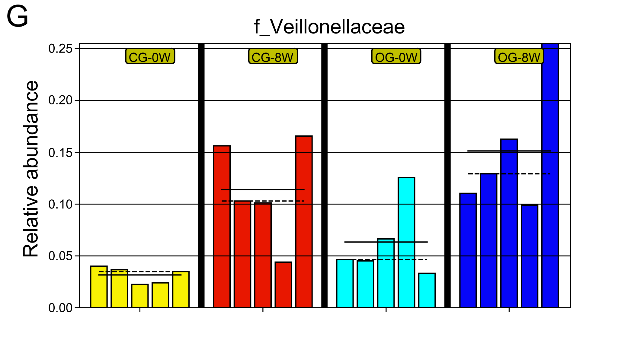


**Figure S2.** Relative abundance on the family and genus level of the microbiomes between the original and control group in 0W and 8W. The straight line is the mean value of relative abundance, and the dot line is the median. Dominant gut bacteria in CG-8W and OG-8W were showed in figures a-d and e-g respectively.
